# The Roles of Tripartite Motif Proteins in Urological Cancers: A Systematic Review

**DOI:** 10.3390/cancers17142367

**Published:** 2025-07-16

**Authors:** Yuta Yamada, Naoki Kimura, Kazuki Maki, Yuji Hakozaki, Fumihiko Urabe, Shoji Kimura, Tetsuya Fujimura, Satoshi Inoue, Haruki Kume

**Affiliations:** 1 Department of Urology, Graduate School of Medicine, The University of Tokyo, Bunkyo-Ku, Tokyo 113-8655, Japan; naoki.kimura331@gmail.com (N.K.); newmakky@gmail.com (K.M.); yhakozaki11012@gmail.com (Y.H.); kumeh-uro@h.u-tokyo.ac.jp (H.K.); 2 Department of Urology, The Jikei University Daisan Hospital, Komae-Shi 201-8601, Tokyo, Japan; furabe0809@gmail.com; 3 Toneri Urology Clinic, Adachi-Ku, Tokyo 121-0831, Japan; shoji.kimura.0221@gmail.com; 4 Department of Urology, Jichi Medical University, Shimotsuke-Shi 329-0498, Tochigi, Japan; tfujimura@jichi.ac.jp; 5 Systems Aging Science and Medicine, Tokyo Metropolitan Institute for Geriatrics and Gerontology (TMIG), Itabashi-Ku, Tokyo 173-0015, Japan; sinoue07@gmail.com

**Keywords:** tripartite motif protein, TRIM, kidney cancer, bladder cancer, prostate cancer, testicular cancer, ubiquitination, urological cancer

## Abstract

We aimed to investigate the roles of tripartite motif (TRIM) proteins in urological cancers. **Methods**: A systematic review was conducted to investigate the oncological role of tripartite motif proteins in urological cancers. **Results**: A total of 84 articles were identified for the final analysis (26 articles on kidney cancers, 19 on bladder cancers, 37 on prostate cancers, and 1 on testicular cancers). In total, 27 TRIM family proteins were involved in kidney cancer, of which 9 were associated with tumor-promoting findings (TRIM24, TRIM27, TRIM37, TRIM44, TRIM46, TRIM47, TRIM59, TRIM63, and TRIM65) and of which 9 TRIM proteins were tumor-suppressive (TRIM2, TRIM7, TRIM8, TRIM13, TRIM21, TRIM26, TRIM28, TRIM33, and TRIM58). Fourteen TRIM family proteins were associated with bladder cancer (tumor-promoting: TRIM9, TRIM25, TRIM26, TRIM28, TRIM29, TRIM59, TRIM65, and TRIM66; tumor-suppressive: TRIM19 and TRIM38). Ten TRIM family proteins were associated with prostate cancer (tumor-promoting: TRIM11, TRIM24, TRIM28, TRIM33, TRIM44, TRIM59, TRIM63, TRIM66, and TRIM68; tumor-suppressive: TRIM32 and TRIM36). Twenty-eight TRIM family proteins were identified to be associated with prostate cancer (tumor-promoting: TRIM11, TRIM24, TRIM28, TRIM33, TRIM44, TRIM59, TRIM63, TRIM66, and TRIM68; tumor-suppressive: TRIM32 and TRIM36). TRIM proteins regulate urological cancers by ubiquitination or modulation of oncologic pathways. **Conclusions**: This review identifies TRIM proteins that are involved in urological cancers. Some of these proteins have the potential to be the therapeutic target.

## Simple Summary

Tripartite motif (TRIM) family proteins are characterized as possessing the RING-finger domain, B-box, and Coiled-coil domain. TRIM proteins are involved in the ubiquitination process and are associated with carcinogenesis. In this study, we performed a systematic review on the functions (tumor promoting or tumor suppressive) of TRIM family proteins in urological cancers. A total of 84 articles were identified for the final analysis and 27, 14, 28, and 1 TRIM proteins were associated with kidney cancers, bladder cancers, prostate cancers, and testicular cancers. TRIM proteins could be a potential therapeutic target for treating cancer and thus this study may give new insights to researchers experimenting on this topic.

## 1. Introduction

The tripartite motif (TRIM) proteins mediate several types of post-translational modifications, most notably, ubiquitination [1,2,3] but also SUMOylation [4,5] and ISGylation [5]. There are over 80 members of the TRIM family in humans, most of which share a common domain structure consisting of a really-interesting-new-gene (RING-finger), B-box, and a coiled-coil domain [1,2,3].

Ubiquitination is a three-step enzymatic cascade that enables degradation of proteins. Ubiquitin binds to the E1 ubiquitin-activating enzyme in an adenosine-triphosphate-dependent (ATP-dependent) manner after it is transferred to an E2 ubiquitin-conjugating enzyme. The ubiquitin is passed on to a lysine residue in a substrate either by direct delivery from E2 that is mediated by the RING type E3 or first transferred to the HECT/RBR type E3 before being transferred to the substrate [1,2,3]. The substrate attached with the ubiquitin chains can be degraded by 26S proteasome. When a single ubiquitin is transferred to a lysine residue of the substrate, it is referred to as “monoubiquitination” [6]. Monoubiquitination can occur at multiple lysine residues, leading to multi-monoubiquitination. Each monoubiquitination can be modified by the formation of polymetric chains containing multiple ubiquitins [7].

SUMOylation and ISGylation are distinct from the ubiquitination process since they are post-translational modifications that use ubiquitin-like proteins, small ubiquitin-like modifier (SUMO) or interferon-stimulated gene 15 (ISG15), instead of ubiquitin and thereby not associated with protein degradation directly. Unlike ubiquitination, these modifications are usually not directly responsible for proteasomal ubiquitination but rather regulated and modified protein activity. Certain types of TRIM proteins are involved in these processes [4,5].

TRIM family proteins function as important regulators in cancer, infectious diseases, and neurogenetic diseases [1,2,3,4,5]. In this review, we focused on the association between TRIM proteins and urological malignancies.

## 2. Materials and Methods

The protocol has been registered in the International Prospective Register of Systematic Reviews database (PROSPERO: CRD42024563339). The Preferred Reporting Items for Systematic Reviews and Meta-Analyses (PRISMA) checklist is reported in Table S1. This study was a systematic review of evaluation on association between urological cancers and TRIM proteins.

## 3. Evidence Acquisition

**Figure 1:**
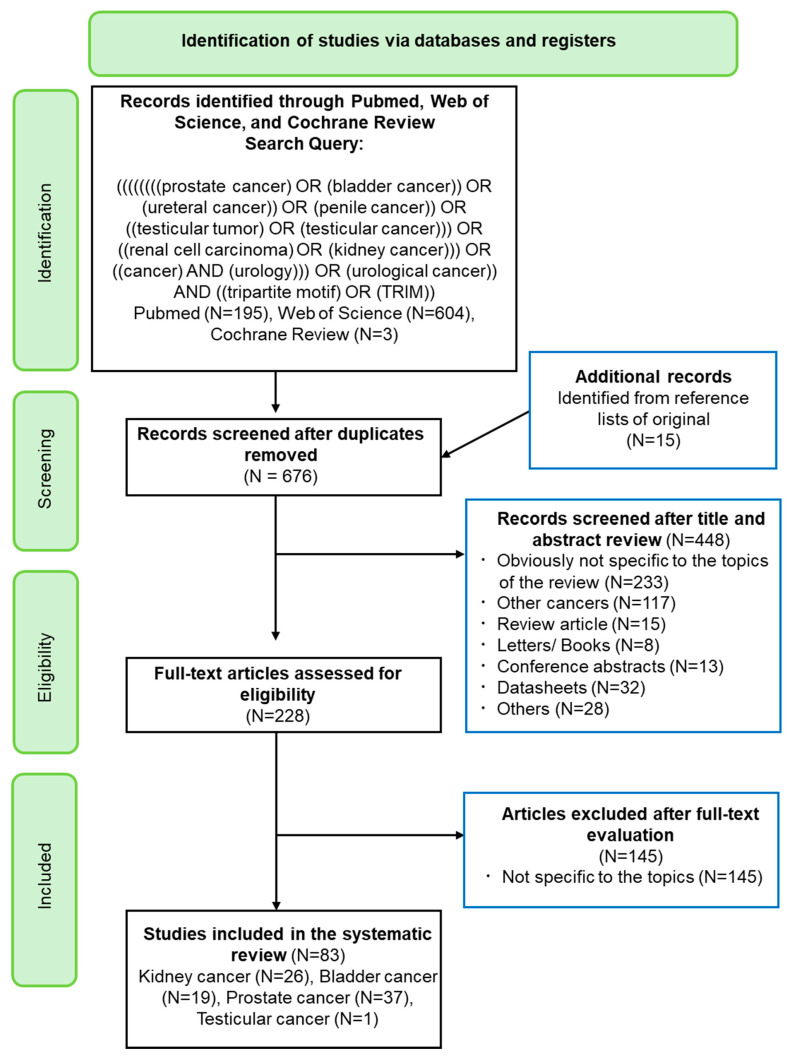
Identification of studies via databases and registers. A total of 83 articles were included for the final review.

Articles presenting association between TRIM proteins and urological cancers were included in this review. Systematic review and meta-analysis were carried out according to the PRISMA statement [8] and the Cochrane Handbook for Systematic Reviews of Interventions [9]. The search of the literature using the electronic databases (Pubmed, Web of Science, Cochrane Library) was performed on 31 May 2024. We first identified potential eligible studies that were appropriate for the topic of this study by performing an initial screening on the titles and abstracts. The review of the literature was conducted using electronic databases (Pubmed, Web of Science, and Cochrane Library) with search combinations of the following keywords in corresponding sections. The keywords ((((((((prostate cancer) OR (bladder cancer)) OR (ureteral cancer)) OR (penile cancer)) OR ((testicular tumor) OR (testicular cancer))) OR ((renal cell carcinoma) OR (kidney cancer))) OR ((cancer) AND (urology))) OR (urological cancer)) AND ((tripartite motif) OR (TRIM)) were used to identify the literature that potentially matched the aim of this review (Figure 1). Representative publications published after year 2000 and other literature relevant to the topic were used in the present review. Studies relevant to human carcinogenesis were included. Conference abstracts, letters/books, datasheets, review articles, articles not written in English, and articles not specific to the topic were excluded (Figure 1). One reviewer (Y.Y.) independently extracted the data, and the second reviewer (F.U.) verified the extracted data. All discrepancies regarding data extraction were resolved by consensus or finally decided by Delphi consensus with other authors. A total of 86 articles, including 27 studies of kidney cancer, 14 studies of bladder cancer, 29 studies of prostate cancer (including CRPC), and 1 study of testicular cancer, were included in the final systematic review (Figure 1).

## 4. Risk of Bias Assessment

The risk of bias and quality of the papers were assessed by the “Risk of Bias In Non-Randomized Studies -of Interventions (ROBINS-I)” for NRCTs (Table S2) [10]. Two authors (Y.Y. and S.K.) independently made assessment of the risk of bias in each study. All discrepancies between the two assessments were resolved by a consensus between the two authors and the supervisor (F.U.).

## 5. Categorization of Classes, Structures of TRIM, and Function of Domains

**Figure 2:**
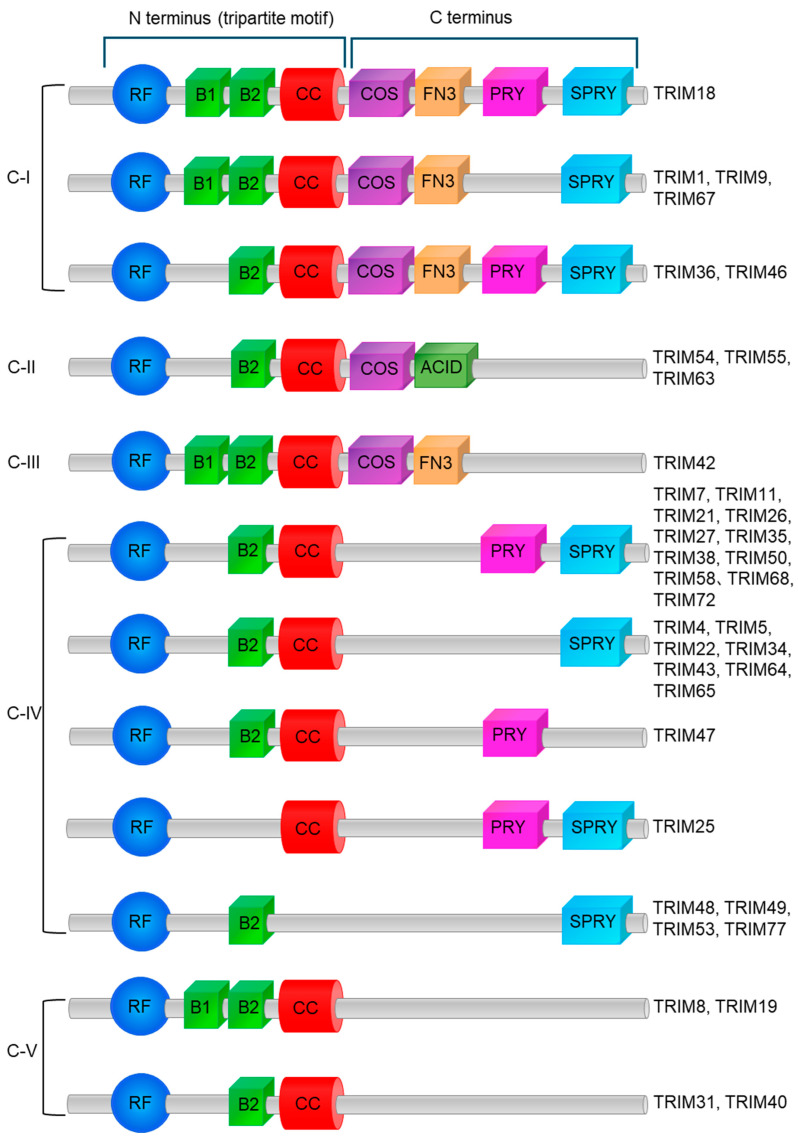

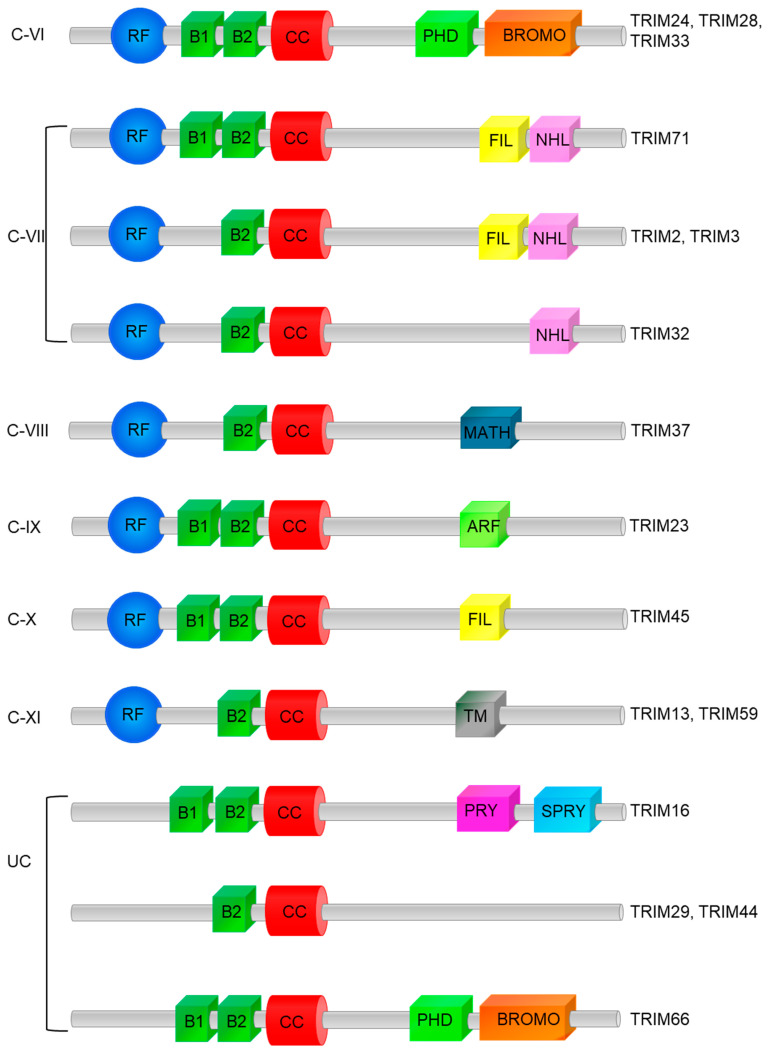
Classification of TRIM protein bases on its structure.

Most TRIM proteins share a conserved N-terminal tripartite motif structure composed of one RING-finger, one or two B-boxes (B-box1 and B-box2), and one coiled-coil domain [1,2,3]. TRIM proteins are further categorized into 11 classes (C-I to C-XI) based on differences in their C-terminal domains [1,2,3]. Not all TRIM proteins have a RING-finger domain and these proteins are unclassified [1,2,3]. Figure 2 shows the classification of the TRIM family proteins that are relevant to urological cancers (Figure 2) [1,2,3].

The B-boxes are also a Cys-His motif much like the RING-finger domain. Each B-box binds one or two zinc ions and is composed of a relatively small number of amino acids. B-box1 of TRIM1 and TRIM18 may contribute to the recognition of the substrate [11]. Other functions of the B-boxes include mediating the protein–protein interactions, including binding to other types of TRIM family members, although their precise biological functions remain unclear. Gushchina et al. hypothesized that B-boxes may have originally evolved as independent E3 ligase domains but evolutionally became functionally secondary to the RING-finger domain [12]. This idea is based on the study that showed the function of B-boxes maintaining the E3 ligase activity of TRIM18, albeit with lower activity than the RING-finger domain [13].

The RING domain forms a RING-finger structure by coordinating two zinc atoms in the middle of the cysteine/histidine residues forming a “cross-brace” structure [14,15]. RING-fingers are capable of acting as E3 ubiquitin ligase and therefore promote conjugation of ubiquitin to a lysine on the target protein by interacting with E2 ubiquitin ligase [1]. Notably, some TRIM proteins (TRIM14, TRIM16, TRIM20, TRIM29, TRIM44, TRIM66, TRIM70, TRIM76, and TRIML1) lack the RING-finger domain [1]. Theoretically, these proteins still have the potential to function as E3 ligases by using the B-boxes, although this theory needs further investigation. Another possible function of these “no-RING” TRIM proteins is the deubiquitination. Taken together, TRIM proteins that lack the RING-finger may retain E3 ligase activity by using B-box domains or may serve alternative roles such as deubiquitination. It is shown that TRIM44 can deubiquitinate p62, an autophagy substrate [16]. Furthermore, TRIM44 can deubiquitinate and stabilize Filamin A (FLNA), an upstream regulator of breast-cancer-1 (BRCA1) [17]. It also binds to other TRIM family members such as TRIM17 and stabilizes it [18].

The coiled-coil domain is responsible for the self-assembly of TRIM proteins and is considered to be involved in homo- and hetero-multimerization of the TRIM proteins (Gushchina, Pharmacol Ther 2018) [12]. This function of higher-order assembly may allow the TRIM protein to provide different tasks depending on the formation of the structure. For instance, TRIM2 and TRIM3 are both members of the C-VII and these proteins are highly homologous in terms of sequence identity in RING-finger and B-box domains [19]. TRIM2 possesses a RING-finger domain that tends to dimerize and form a higher order self-association while the RING-finger domain of the TRIM3 is monomeric and does not develop high-order self-association form [19]. Interestingly, these TRIM proteins have different functional roles despite their similarity of molecular structure. TRIM2 is neuroprotective and ubiquitinates Bcl-2-interacting mediator of cell death (Bim) during rapid ischemic tolerance in the nervous system [20]. This protein also promotes cancer cell proliferation, migration, and invasion by deubiquitinating Snail1 in lung adenocarcinoma [21]. In contrast, TRIM3 polyubiquitinates γ-actin and controls hippocampal plasticity and learning [22].

## 6. The Role of TRIM Proteins in Kidney Cancer

A total of 27 TRIM family proteins were cancer-associated, of which 9 were associated with oncogenic findings (TRIM24, TRIM27, TRIM37, TRIM44, TRIM46, TRIM47, TRIM59, TRIM63, and TRIM65) and 9 TRIM family proteins (TRIM2, TRIM7, TRIM8, TRIM13, TRIM21, TRIM26, TRIM28, TRIM33, and TRIM58) were associated with tumor-suppressive evidence (Table 1) [23,24,25,26,27,28,29,30,31,32,33,34,35,36,37,38,39,40,41,42,43,44,45,46,47,48]. TRIM19 was the only TRIM protein to have reported both oncogenic and tumor-suppressive effects (Table 1) [31,32]. The most common pathways regulated by TRIM family proteins were TGFβ pathway, PI3K/AKT/mTOR pathway, and epithelial–mesenchymal transition (EMT)-related pathway.

TRIM7, TRIM13, and TRIM26 regulate the PI3K/AKT/mTOR pathway as tumor suppressors in kidney cancers [27,30,36]. TRIM7 inhibits Hypoxia Inducible Factor 1 alpha (HIF-1ɑ) by targeting Src protein and regulating PI3K/AKT/mTOR pathway [27]. Upregulation of TRIM13 inhibits NFκβ, MMP-9, and p-AKT and suppresses cell migration and invasion [30], while TRIM26 degrades ETK and inhibits the AKT/mTOR pathway [36].

TRIM family proteins, which act as tumor promoters, include TRIM24 [28,35], TRIM27 [24,26,28], TRIM37 [41], TRIM44 [42], TRIM46 [43], TRIM47 [44], TRIM58 [26,45], TRIM59 [46], TRIM63 [47], and TRIM65 [26,48]. Among these proteins, TRIM24 and TRIM37 promoted EMT and facilitated cell proliferation and invasion [34,41]. Notably, TRIM33 is controversial regarding its function involved with cancer progression. Xu et al. investigated a Cancer Genome Atlas (TGCA) database and found that mRNA expression of TRIM33 in clear cell renal carcinoma tissues were downregulated and that low TRIM33 expression was associated with poor prognosis [40]. Gain-of-function study in 786-O and ACHN kidney cancer cells lines also showed that TRIM33 overexpression inhibited cell proliferation, migration, and invasion of these cancer cells [40].

TRIM44, a known tumor-suppressing gene, downregulates Fyn-related kinase (FRK) and promotes cancer progression in renal cell carcinoma [42]. The downregulation of FRK by TRIM44 can also be observed in ovarian cancer [49]. In fact, TRIM44 is associated with tumor progression in over 10 types of cancer, including thyroid cancer [50,51], esophageal cancer [52,53,54], gastric cancer [55,56,57], lung cancer [58,59,60,61,62], hepatocellular carcinoma [63], colorectal cancer [64,65,66], ovarian cancer [67,68,69], cervical cancer [70], breast cancer [71], prostate cancer [72,73], and testicular cancer [74]. Genomic and transcriptomic data revealed a TRIM44 overexpression in 16.1% of epithelial cancers and a positive correlation between TRIM44 and mTOR signaling [75]. Previous reports identified NFκβ signaling pathway as a target pathway of TRIM44 in cancers of the larynx [76], lung [59], liver [63], breast [71], colorectal [66], and ovary [69]. Other pathways regulated by TRIM44 included the AKT-related pathway in esophagus [52], non-small cell lung cancer (NSCLC) [60], colorectal [64], prostate [72], and glioma cancers [77]. TRIM44 is also among the downstream targets of circRNA in regulating glycolysis in cancer [78].

The role of tumor-suppressor protein promyelocytic leukemia (PML), also known as TRIM19, is controversial regarding the involvement with cancer progression. Both mRNA expression and protein levels of PML were overexpressed based on the data of The Cancer Genome Atlas (TCGA) and National Cancer Institute’s Clinical Proteomic Tumor Analysis Consortium (CPTAC) [28,32]. Simoni et al. showed that PML knockdown leads to accumulation of G0/G1 distribution in RCC cell lines and inhibition of tumor growth in vivo [32]. PML knockdown also promoted accumulation of p53 and its bona fide transcriptional target p21 for growth arrest in clear cell RCC cell lines [32]. In contrast, Lin et al. revealed a mechanism involving the stabilization of PML by SCP phosphatases and inhibition of mTOR/HIF signaling [31]. They found that phosphatase SCP1 and its isoforms SCP2/3 dephosphorylate PML at S518 and block the degradation of PML [31]. SCP1 stabilized PML, which led to enhanced tumor-suppressive effects regarding proliferation, migration, invasion tumor growth, and tumor angiogenesis. Furthermore, it suppressed the mTOR-HIF pathway [31].

## 7. The Role of TRIM Proteins in Bladder Cancer

Bladder cancer is characterized by a high potential tumor recurrence. As shown in Table 2, 14 TRIM family proteins were reported to have association in carcinogenesis [79,80,81,82,83,84,85,86,87,88,89,90,91,92,93,94,95,96,97]. Among them, TRIM9 [79], TRIM25 [88], TRIM26 [89], TRIM28 [87], TRIM29 [90], TRIM59 [93,94], TRIM65 [95], and TRIM66 [96] were previously reported as having oncogenic association with bladder cancer. Several pathways, including the CEACAM6-SMAD2/3 pathway [79], NF-kappa beta pathway [86], and AKT pathway [86,89], were associated with TRIM proteins that promoted cancer progression of bladder cancer. Notably, TRIM24 and TRIM26 both activate the AKT pathway and promote cell proliferation and invasion [86,89]. TRIM59 [94] and TRIM65 [95] modulated epithelial–mesenchymal transition of urothelial carcinoma cells by ubiquitination of target proteins. Other TRIM proteins (TRIM9, TRIM38, TRIM65, and TRIM66) promoted progression of bladder cancer by targeting GLUT1 [92], ANXA2 [95], and MMP11 [96].

Chen W et al. found that TRIM59 is associated with promotion of EMT and promotion of cell proliferation, invasion, and migration via activation of transforming growth factor TGF-beta/SMAD2/3 signaling pathway [93]. Another study investigated the stiffness of bladder cancer cells by using atomic force microscopy. Tumor cells with intrinsic softness potentially function as cancer stem cells in various types of cancer [93]. Consequently, bladder cancer, of which intrinsic soft tumor cells are identified, is known to be associated with cancer recurrence [93]. They found that ITGB8/TRIM59/AKT/mTOR/glycolysis pathways were upregulated in bladder cancers with soft tumor cells [93].

Multiple studies have shown that TRIM21 is associated with bladder cancer [83,84,85]. TRIM21 binds to Prothymosin Alpha (PTMA) and degrades it, reducing both nuclear and cytoplasmic PTMA [83]. This reduction in PTMA levels leads to decreased PTEN expression and enhanced Nrf2 signaling, both of which show oncogenic effect [83]. TRIM21 also removes the inhibitory effect that PTMA has on Nuclear Respiratory Factor 1 (NRF1) signaling by degrading PTMA [83]. Another study revealed that TRIM21 is also associated with the interaction with ubiquitin-conjugating E2 enzyme (UBE2S) that leads to the ubiquitination of lipoma preferred partner (LPP) via K11-linked polyubiquitination, thereby promoting the lymphatic metastasis of bladder cancer [85]. The authors further showed that targeting UBES with cephalomannine inhibited the progression of bladder cancer cells and human bladder-cancer-derived organoids in vitro and lymphatic metastasis model in vivo [85]. In contrast, the research by Deng M et al. discovered that Zinc Fingers And Homeoboxes 3 (ZHX3), a known oncogenic factor associated with poor prognosis, was a target of degradation by TRIM21 [84]. Taken together, TRIM21 changes its oncogenic and tumor-suppressive roles depending on its binding targets.

## 8. The Role of TRIM Proteins in Prostate Cancer

A total of 29 TRIM family proteins are involved in prostate cancer (Table 3) [72,73,98,99,100,101,102,103,104,105,106,107,108,109,110,111,112,113,114,115,116,117,118,119,120,121,122,123,124,125,126,127,128,129,130]. TRIM family proteins regulate various pathways such as AKT/PI3K pathway, apoptosis-related pathway, glycolysis pathway, and HIF-1 pathway (Table 3).

Among the TRIM family proteins regulating carcinogenesis in prostate cancer, TRIM36 is a unique protein since this protein is both androgen-dependent and also acts as a tumor suppressor [122]. TRIM36 was initially identified as haprin, which was involved in the acrosome reaction [131]. Fujimura et al. investigated mRNA expression of both cancerous and stromal tissues of biopsy samples and identified TRIM36 as one of the 10 genes associated with cancer tissues [121]. Low expression of TRIM36 level is also observed in neuroendocrine prostate cancer and TRIM36 inhibits the glycolysis pathway by downregulating Glutathione Peroxidase 4 (GPX4) by interacting with lys-48 and HK2 [123].

TRIM25, also known as estrogen-responsive finger protein (Efp), is an estrogen-induced protein that is involved in promoting cancer progression of cancer originating in prostate as well as in breast [132], liver [133], and in colon [134]. In prostate cancer, TRIM25 stabilizes the TP53/G3BP2/RanBP2 complex, which inhibits the tumor-suppressive function of TP53 in the nucleus [113]. TRIM25 also targets Erg, a known driver of prostate carcinogenesis, and degrades it [112]. TRIM25 not only targets the 14-3-3 sigma, a known tumor suppressor of prostate cancer, for degradation by ubiquitination but it also mediates ISG15 modification of 14-3-3 sigma [135].

TRIM68 is another TRIM protein that is androgen-dependent and interacts with androgen receptor (AR) in the presence of dihydrotestosterone [128]. TRIM68 interacts with coactivators of AR such as TIP60 and p300 to mediate AR transcription in prostate cancer [128]. Li et al. discovered two micro RNAs (miR-29a and miR-1256) that directly target and regulate TRIM68 to inhibit prostate cancer cell growth and invasion [129]. Nie et al. found YTHDF1 as an upper stream regulator of TRIM68 by investigation with m6A RNA immunoprecipitation sequencing and bioinformatics analysis [130]. Together with the RNA binding protein GAP SH3 Binding Protein (G3BP1), YTH N6-Methypadenosine RNA Binding Protein F1 (YTHDF1) is involved in regulating AR mRNA translation [136]. Intriguingly, YTHDF1 is also an upper stream regulator of TRIM44 in prostate cancer [73].

## 9. The Role of TRIM Proteins in Castration-Resistant Prostate Cancer

Castration-resistant prostate cancer (CRPC) is a condition in which androgen deprivation treatment no longer is effective. The cancer-specific survival of non-metastatic CRPC patients was 38 months [137]. Metastatic-CRPCs are much lethal, and the median overall survival is 23 months [138].

TRIM24, also known as Transcriptional Intermediary Factor 1 (TIFɑ), is a transcriptional coactivator of androgen receptor (AR) and an oncogenic member of the TRIM family. Bai et al. identified a post-transcriptional regulatory mechanism of TRIM24, whereby the LINC00963 promotes TRIM24 expression and cell proliferation in CRPC cells in vitro and in vivo by directly binding to miR-655, which is an inhibitor of TRIM24 [110]. In parallel, TRIM24 is also subject to post-translational regulation through ubiquitination by the E3 ubiquitin ligase adaptor SPOP [116]. Fong et al. revealed that TRIM28 binds to TRIM24 and protects it from SPOP-mediated ubiquitination, thereby stabilizing TRIM24 and consequently promoting cell proliferation and tumor growth in vitro and in vivo [116]. Taken together, these studies indicate a multilayered regulation of TRIM24 at both post-transcriptional and post-translational levels, highlighting its important oncogenic role in CRPC.

Kimura et al. investigated the function of TRIM36 in CRPC cells [122]. Knockdown of TRIM36 suppressed apoptosis and promoted cell proliferation and migration in 22Rv1 cells and vice versa for TRIM36 overexpression [122]. Additionally, the knockdown of TRIM36 alleviated docetaxel-induced apoptosis in 22Rv1 cells, suggesting that TRIM36 plays a significant role in regulating chemotherapy response [122].

TRIM59, which has an oncogenic role in both kidney cancers [46] and bladder cancers [95,98], also has an oncogenic activity in CRPC [125]. TRIM59 is highly expressed in CRPC and is associated with poor prognosis. The AR directly binds to the TRIM59 promoter region, repressing its transcription [125]. Upon AR inhibition, this repression is lifted, resulting in promotion of TRIM59 expression. Functional studies revealed that knockdown of TRIM59 inhibited CRPC cell proliferation, migration, and tumor growth [125]. The study further demonstrated that TRIM59 promotes degradation of tumor suppressor proteins RB1 and p53, which leads to increased SRY-box Transcription Factor 2 (SOX2) expression and drives neuroendocrine differentiation in prostate cancer [125].

## 10. The Role of TRIM Proteins in Testicular Cancer

There is only one paper describing the impact of TRIM protein in testicular cancer [74]. TRIM44 overexpression was associated with alpha feto protein levels, clinical stage, nonseminomatous germ cell tumor, and cancer-specific survival [74]. TRIM44 upregulated Complement Component 3a (C3AR1), ST3 Beta-Galactoside Alpha-2,3-Sialytransrefase 5 (ST3GAL5), and Ecto-5′-Nucleaotidase (NT5E), while it downregulated Cell Adhesion Molecule 1 (CADM1), Cyclin-Dependent Kinase 19 (CDK19), and Protein Kinase CAMP-Activated Catalytic Subunit Beta (PRKACB) that lead to promoting cell proliferation and migration of NTERA2 and NEC8 cells [74].

## 11. The Role of TRIM Proteins in Other Types of Urological Malignancies

Unfortunately, there is no report describing the association between TRIM proteins and upper urinary tract urothelial carcinoma and penile cancer.

## 12. Conclusions

We performed a systematic review on the oncological function of the TRIM proteins. There are approximately 80 types of TRIM proteins, of which some may be a good potential therapeutic target for treating cancer. Future experimental studies on testicular cancer and upper urinary tract cancer are required due to a small number of studies.

## Figures and Tables

**Table 1:** Articles describing association of TRIM proteins with kidney cancer (N = 26).

Authors	Journals, Year	TRIM	Tumor-Promoting (P)/ Tumor-Suppressive (S)	Involved Signal/ Pathway	Summary
Zheng [23]	Front Oncol, 2022	TRIM1, TRIM2, TRIM13, TRIM26, TRIM27, TRIM35, TRIM47, TRIM55	NA	NA	Prognostic signature was developed using 8 TRIM family proteins. Higher risk scores were associated with higher level of immune infiltration by plasma cells, follicular helper T cells, and NK cells and a lower level of immune infiltration by memory resting CD4 T cells, M1 and M2 macrophages, and resting dendritic cells.
Xiao [24]	Cancer Manag Res, 2018	TRIM2	S	NA	Low expression of TRIM2 was correlated with poor prognosis. Overexpression of TRIM2 promoted cell proliferation, migration, and invasion in RCC cell line.
Wei [25]	Aging, 2020	TRIM2	S	NA	TRIM2 was related to clinical stage and pathological grade and was an independent prognostic factor. KEGG and GO indicated ubiquitin mediated protein hydrolysis, cell adhesion molecules, and Th17 cell differentiation signaling pathway.
Shen [26]	Aging, 2022	TRIM4, TRIM7, TRIM27, TRIM58, TRIM65, TRIM72	NA	NA	These TRIM proteins showed positive correlation with worse survival in kidney clear cell carcinoma patients.
Yuan [27]	Cell Biology Int, 2022	TRIM7	S	HIF-1ɑ, Src; PI3K/AKT/mTOR pathway	TRIM7 acts as a tumor suppressor which inhibits HIF-1ɑ through degrading Src protein and regulating PI3K/AKT/mTOR pathway or reactive oxygen species production.
Wu [28]	Int J Med Sci, 2020	TRIM8, TRIM11, TRIM16, TRIM19, TRIM27	NA	NA	high mRNA expression levels of these genes were correlated with poor prognosis.
		TRIM24, TRIM32	NA	NA	Low mRNA expression levels of these genes were correlated with poor prognosis.
Caratozzolo [29]	Oncotarget, 2014	TRIM8	S	p53	TRIM8 upregulation restores p53 tumor suppressor response to chemotherapeutic drug treatments in renal cell carcinoma cell lines.
Li [30]	Nutr Cancer, 2020	TRIM13	S	NFκβ, MMP-9, p-AKT	Upregulation of TRIM13 inhibited NFκβ, MMP-9, and p-AKT and suppressed cell migration and invasion in RCC cell line.
Lin [31]	Cancer Res, 2014	TRIM19	S	SCP1; mTOR-HIF pathway	SCP1 inhibits the degradation of PML that inhibits the cell proliferation, migration, invasion, angiogenesis, tumor growth, and mTOR-HIF pathway in clear cell renal cell carcinoma.
Simoni [32]	EMBO Mol Med, 2024	TRIM19	P	p53, cell cycle	PML knockdown leads to accumulation of G0/G1 distribution in RCC cell lines and inhibition of tumor growth in vivo.
Chen [33]	Cancers Letters, 2021	TRIM21	S	HIF-1α	TRIM21 inhibits RCC cell glycolysis via the ubiquitination-mediated degradation of HIF-1ɑ leading to inhibition of cell proliferation, tumorigenesis, migration, and metastasis of RCC cells in vitro and in vivo.
Jiang [34]	Open Medicine, 2020	TRIM24	P	MMP-2, MMP-9, fibronectin, SNAIL, vimentin, N-cadherin, β-catenin; EMT	TRIM24 promoted cell proliferation, migration, and invasion of RCC cell line.
Yu [35]	Cancer Sci, 2020	TRIM24	P	Wnt/beta catenin pathway	TRIM24 promoted the invasion and migration of clear cell RCC cells through the Wnt/beta catenin pathway.
Zheng [36]	J Transl Med, 2024	TRIM26	S	ETK, AKT/mTOR	Low expression of TRIM26 is associated with worse overall survival. TRIM26 inhibits cell proliferation, migration, and invasion by degrading ETK resulting in inhibition of AKT/mTOR signaling pathway in clear cell RCC cel line.
Xiao [37]	BMC Cancer, 2021	TRIM27	P	lκβα	High expression of TRIM27 was correlated with poor prognosis. TRIM27 interacted with lκβɑ that promoted NFκβ.
Song [38]	J Biol Chem, 2023	TRIM28	S	TFE3, histone H3K27 demethylase KDM6A	TRIM28 inhibits TFE3 that interacts with and recruits histone H3K27 demethylase KDM6A for autophagic gene upregulation and suppresses tumor growth.
Jingushi [39]	Mol Cancer Res, 2015	TRIM33	S	miR-629, TGF-β; SMAD signaling, EMT	miR-629 inhibits TGF-beta-induced SMAD activation via upregulation of TRIM33.
Xu [40]	Biomed Res Int, 2020	TRIM33	S	β-catenin, cyclin D1, c-myc	Low expression of TRIM33 was related to poor prognosis. TRIM33 overexpression inhibited cell proliferation, migration, and invasion of RCC cell lines and reduced β-catenin, cyclin D1, and c-myc, and inhibited tumor growth in vivo.
Miao [41]	J Exp Clin Cancer Res, 2021	TRIM37	P	TGF-β1 signaling, EMT	TRIM37 promoted ubiquitination of histone H2A and promoted EMT and cancer progression through activating TGF-β1 signaling.
Yamada [42]	Cancer Sci, 2020	TRIM44	P	FRK	TRIM44 overexpression was associated with clinical M stage, clear cell type, lymphatic invasion, and cancer-specific survival. TRIM44 promotes cell proliferation via regulating FRK in RCC cell line.
Ren [43]	Front Med, 2021	TRIM46	P	tumor immunity several pathways	TRIM46 upregulation was associated with unfavorable prognosis based on the bioinformatics analyses using the data from TCGA and GEO databases. TRIM46 was positively correlated with NUMBL, CACNB1, THBS3, ROBO3, MAP3K12, ANKRD13B, and PCNX2.
Chen [44]	Cancer Cell Int, 2021	TRIM47	P	p53	TRIM47 promoted RCC proliferation in vitro and in vivo via degrading p53 by ubiquitination.
Gan [45]	Front Mol Biosci, 2021	TRIM58	S	NA	TRIM58-specific DNA demethyltransferase promotes demethylation of TRIM58 CpG islands and activates the TRIM58 transcription that leads to inhibition of cell proliferation and migration in RCC cell lines.
Hu [46]	Cell Mol Biol, 2017	TRIM59	P	NA	TRIM59 knockdown inhibited cell migration in 786-O cells line and inhibited tumor growth in vivo.
Wang [47]	Mod Pathol, 2021	TRIM63	P	TFE3	TRIM63 was highly expressed in MiT family aberration-associated RCC. TRIM63 RNA-ISH was strongly positive in TFE3 FISH false-negative cases.
Zhang [48]	Cell Death Dis, 2024	TRIM65	P	BTG3; cell cycle	TRIM65 promoted cell proliferation by degrading BTG3 and suppressing G2/M phase cell cycle arrest.

NA: data not available, TRIM: tripartite motif, RCC: renal cell carcinoma, KEGG: Kyoto Encyclopedia of Genes and Genomes, GO: gene ontology, EMT: epithelial–mesenchymal transition, TCGA: The Cancer Genome Atlas, GEO: Gene Expression Omnibus.

**Table 2:** Articles describing association of TRIM proteins with bladder cancer (N = 19).

Authors	Journals, Year	TRIM	Tumor-Promoting (P)/ Tumor-Suppressive (S)	Involved Signal/ Pathway	Summary
Zhang [79]	J Cell Commun Sigaling, 2023	TRIM9	P	CEACAM6	TRIM9 promoted cell proliferation and migration of bladder cancer cells. TRIM9 modulated CEACAM6 upregulation, which facilitated Smad2/3-MMP2 signaling pathway in vitro and in vivo. Overexpression of TRIM9 reduced the chemosensitivity to mitomycin C and gemcitabine in bladder cancer cell line.
He [80]	Chin Med J, 2003	TRIM19	S	NA	PML inhibited cell proliferation by inducing G1 cell cycle arrest and apoptosis in vitro and in vivo.
Xue [81]	Oncol Rep, 2010	TRIM19	S	p53	Inhibition of teromerase activates p53 via PML regulation.
Li [82]	Cancer Lett, 2006	TRIM19	S	Caspase-dependent pathway	PML induced apoptosis of bladder cancer cell by promoting Caspase-dependent pathway.
Tsai [83]	Cancer Sci, 2019	TRIM21	S	PTEN, Nrf2 signaling	PTMA upregulates PTEN and interacts with TRIM21 to regulate Nrf2 signaling.
Deng [84]	Cancer Sci, 2021	TRIM21	S	ZHX3	TRIM21 regulates ZHX3, which inhibits RGS2.
Xiao [85]	Cell Death Dis, 2023	TRIM21	P	UBE2S	UBE2 interacting with TRIM21 degrades LPP through k11-linked ubiquitination and promotes the lymphatic metastasis of bladder cancer.
Xue [86]	Tumour Biol, 2015	TRIM24	P	NFκβ, AKT pathway	TRIM24 promotes cell proliferation and invasion. TRIM24 upregulated cyclinD1, cyclinE, p-lκβα, and p-AKT.
Agarwal [87]	Proc Natl Acad Sci U S A, 2021	TRIM24	S	TRIM28	TRIM28 activates hTERT and promotes bladder cancer cell growth. TRIM24 interacts with TRIM28 and inhibits its activity.
		TRIM28	P	hTERT	
Tang [88]	Cancer Sci, 2022	TRIM25	P	RBPJ	RITA1 promotes bladder cancer cell proliferation by interacting with TRIM25 and degrading RBPJ.
Xie [89]	Chem Biol Interact, 2021	TRIM26	P	p-AKT, p-GSK3β, β-catenin, c-Myc	TRIM26 upregulates bladder cancer cells via the AKT/GSK3β/β-catenin pathway.
Palmbos [90]	Cancer Res, 2015	TRIM29	P		TRIM29 is highly expressed in huma bladder cancer and correlates with invasive disease. TRIM29 promotes cell proliferation and invasion.
Zhang [91]	Oncol Lett, 2017	TRIM31	NA	NA	TRIM31 as well as LGALS4, PTPRN2, TMPRSS11E, and KCND3 were identified as 5 hub genes associated with bladder cancer.
Wang [92]	J Transl Med, 2021	TRIM38	S	GLUT1	TRIM38 degrades GLUT1 and act as tumor suppressor in vitro and in vivo
Chen [93]	Onco Targets Ther, 2017	TRIM59	P	TGF-β/Smad2/3 signaling pathway	TRIM59 induced EMT via activation of TGFβ/Smad2/3 signaling pathway.
Qiu [94]	Chinese Med J, 2024	TRIM59	P	NA	3D Matrigel activated the F-actin/ITGB8/TRIM59/AKT/mTOR/glycolysis pathways to promote softness of tumor cells.
Wei [95]	Cancer Lett, 2018	TRIM65	P	ANXA2, EMT	TRIM65 induces EMT of urothelial carcinoma of the bladder cells by degrading ANXA2.
Xiao [96]	J Biomed Nanotechnol, 2023	TRIM66	P	MMP-11	Knockdown of TRIM66 inhibits bladder cancer cell proliferation and migration by downregulating MMP-11.
Chen [97]	BMC Cancer, 2021	TRIM71	NA	NA	Higher mRNA expression level of TRIM71 based on the RNA sequence data from TCGA database.

NA: data not available, TRIM: tripartite motif, EMT: epithelial–mesenchymal transition, TCGA: The Cancer Genome Atlas.

**Table 3:** Articles describing association of TRIM proteins with prostate cancer (PCa), including castration-resistant PCa and neuroendocrine PCa (N = 37).

Authors	Journals, Year	TRIM	Tumor-Promoting (P)/ Tumor-Suppressive (S)	Involved Signal/ Pathway	Summary
Offermann [98]	Carcinogenesis, 2021	TRIM1, TRIM5, TRIM21, TRIM23, TRIM33, TRIM36, TRIM44, TRIM4, TRIM10, TRIM40, TRIM42, TRIM50, TRIM66, TRIM67, TRIM71, TRIM77	NA	TNF, TGF-β, PI3K/AKT, HIF-1 signaling pathway	Transcriptome analysis was carried out in 59 patients, including localized and bone metastatic prostate cancer. A total of 7 TRIM genes (TRIM1, TRIM5, TRIM21, TRIM23, TRIM33, TRIM36, and TRIM44) were lower expressed and 9 TRIM genes (TRIM 4, TRIM10, TRIM40, TRIM42, TRIM50, TRIM66, TRIM67, TRIM71, and TRIM77) were overexpressed in bone metastatic prostate cancer compared to localized cancer.
Pan [99]	Med Sci Monit, 2019	TRIM11	P	NA	TRIM11 was upregulated in prostate cancer tissue and was associated with poor prognosis. TRIM11 overexpression promoted cell proliferation and was inhibited by miR-5193.
Pan [100]	Technol Cancer Res Treat, 2023	TRIM11	P	MEK1/2, ERK1/2	miR-5193 suppresses cell proliferation by inhibiting TRIM11 and MEK1/2 and ERK1/2 pathway in vitro. miR-5193 also inhibited tumor growth in vivo by modulating TRIM11.
Guo [101]	Crit Rev Eukaryot Gene Exp, 2022	TRIM11	P	FAM46B	TRIM11 was upregulated in paclitaxel-resistant cells and inhibited FAM46B that led to promotion of cell proliferation, migration, and invasion in these cells.
Spirina [102]	Asian Pac J Cancer Prev, 2020	TRIM16	NA	NA	Higher expression of progesterone receptor (PR) was observed in cancer tissues. Low PR level was associated with high expression of TRIM16.
Buczek [103]	Oncogene, 2016	TRIM19	P	TGF-β, SMAD2/3; EMT	Cytoplasmic PML promotes EMT by activating TGF-β canonical signaling pathway through the induction of SMAD2/3 phosphorylation.
Birch [104]	Ann Oncol, 2014	TRIM19	NA	NA	Low expression levels of PML protein were associated with cancer-specific death.
Chatterjee [105]	Cell Death Dis, 2013	TRIM19	S	CK2, PHLPP2, FOXO3a, pAKT; AKT pathway	Inhibition of CK2 promotes PML elevation, which interacts with PHLPP2 in the nucleus and increases FOXO3a activity, leading to inhibition of cell proliferation and promotion of apoptosis in prostate cancer cells.
Zhang [106]	Cancer Immun, 2003	TRIM19	NA	NA	Low expression levels of PML protein were observed in prostate cancer tissue by IHC.
He [107]	Cancer Res, 1997	TRIM19	S	NA	PML suppresses tumor growth in prostate cancer cells
Yang [108]	Biochem Biophys Res Commun, 2004	TRIM19	S	p21, p53, AR	PML inhibits androgen receptor (AR) transactivation and promotes p21 and p53 activity. Knockdown of PML promotes AR activity and cell proliferation.
Höflmayer [109]	Appl immunohistochem Mol Morphol, 2021	TRIM24	NA	NA	TRIM24 upregulation was associated with high Gleason grade, advanced pathological T stage, lymph node metastasis, higher preoperative PSA level, increased cell proliferation, and genomic instability.
Bai [110]	Front Oncol, 2021	TRIM24	P	miR-655, Linc00963	Lin00963 interacts with miR-655 and upregulates TRIM24, thereby activating PI3K/AKT signaling and promoting cell proliferation in CRPC cells.
Guan [111]	Am J Trans Res, 2019	TRIM24	P	MeCP2, DNMT, miRNa-137; glutamine metabolism	MeCP2 and DNMTs interacted to promote epigenetic silencing of miRNA-137, which led to promotion in TRIM24 upregulation and glutamine metabolism in bicalutamide-resistant prostate cancer cells.
Wang [112]	Oncotarget, 2016	TRIM25	S	ERG	TRIM25 degrades ERG by ubiquitination.
Takayama [113]	Oncogene, 2018	TRIM25	P	G3BP2	TRIM25 promoted cell proliferation and inhibited apoptosis by modulating p53 signals via regulation of G3BP2.
Li [114]	Int J Mol Sci, 2022	TRIM25	NA	IDH1, FH	TRIM25 is involved in glucose metabolism by regulating IDH1 and FH. TRIM25 expression level was positively associated with Gleason scores.
Yu [115]	Cancer Biother Radiopharm, 2022	TRIM25	P	SNHG3, miR-487	SNHG3 mediates migration, invasion, and EMT in Pca cells by sponging miR-487 a-3p to regulate TRIM25.
Fong [116]	Nat Commun, 2018	TRIM28	P	TRIM24	TRIM28 stabilizes TRIM24 by blocking SPOP-mediated degradation and promotes CRPC tumor growth.
Xue [117]	FASEB J, 2024	TRIM28	P	T cell immune system	Knockdown of TRIM28 reduces proportions of M2 macrophages, enhanced infiltration of CD8+ T cells, and reduced tumor growth of CRPC cells.
Kanno [118]	Acta histochem, 2014	TRIM29	NA	NA	TRIM29 is selectively expressed in basal cells of normal prostate.
Zhou [119]	J Exp Clin Cancer Res, 2023	TRIM32	S	TSPAN18, STIM1	TSPAN18 promotes bone metastasis by interacting with STIM1 that blocked from TRIM32-mediated ubiquitination and degradation.
Chen [120]	EMBO Rep, 2022	TRIM33	P	AR	TRIM33 promotes tumor growth by stabilizing AR from Skp-mediated degradation in prostate cancer cells.
Fujimura [121]	Clin Cancer Res, 2014	TRIM36	NA	NA	Lower mRNA expression of TRIM36 was correlated with lower cancer-specific survival.
Kimura [122]	Cancer Sci, 2018	TRIM36	S	BAX, TNFSF10; apoptosis-related pathway, T-cell receptor signaling pathway	High expression of TRIM36 is associated with favorable prognosis. TRIM36 promotes apoptosis and suppresses cell proliferation and migration in prostate cancer cell lines. TRIM36 acts as tumor suppressor by upregulating BAX and TNFSF10.
Zhao [123]	Cancer Sci, 2023	TRIM36	S	lys-48, HK2, GPX4; glycolysis pathway	TRIM36 was expressed low in neuroendocrine prostate cancer. TRIM36 inhibited the glycolysis pathway by promoting K48-linked ubiquitination of HK2. This loss of HK2 activity leads to downregulation of GPX4, which then makes the cancer cells vulnerable to ferroptosis.
Tan [72]	Oncol Res, 2017	TRIM44	P	PI3K/AKT pathway	TRIM44 was upregulated in prostate cancer cell lines. Knockdown of TRIM44 inhibited proliferation and invasion of prostate cancer cells in vitro and reduced tumor growth in vivo. Knockdown of TRIM44 also reduced the levels of phosphorylated PI3K and Akt in PC-3 cells.
Li [73]	Genes Genomics, 2021	TRIM44	P	YTHDF1	YTHDF1 promotes cell proliferation, migration, and invasion by modulating TRIM44.
Zhou [119]	J Biochem Mol Toxicol, 2023	TRIM44	P	NA	Knockdown of long non-coding RNA, LINC00491, inhibited cell proliferation in vitro and reduced tumor growth in vivo. LINC00491 is a tumor promoter that upregulates TRIM44 by sponging miR-384 to facilitate cancer progression.
Fujimura [121]	Clin Genitourin Cancer, 2016	TRIM47	NA	NA	Based on immunohistochemical findings, high expression levels of TRIM47 were associated with ≥pT3 stage and worse cancer-specific survival.
Lin [124]	Eur Rev Med Pharmacol Sci, 2016	TRIM59	P	CDC25A, CDC2, cyclin B1; cell cycle	TRIM59 was highly expressed in prostate cancer tissues. TRIM59 promotes cell proliferation by upregulating CDC25A, CDC2, and cyclin B1.
Fan [125]	Oncogene, 2023	TRIM59	P	AR, RB1, p53	AR inhibits TRIM59, which is highly expressed in CRPC. TRIM59 is associated with poor prognosis. TRIM 59 knockdown suppresses CRPC cell proliferation, migration, and tumor growth in vitro and in vivo. TRIM59 promotes degradation of RB1 and p53.
Ma [126]	Tropical J Pharma Res, 2022	TRIM63	P	microRNA-300	microRNA-300 suppresses cell proliferation and migration by targeting TRIM63.
Cao [127]	FEBS Open Bio, 2020	TRIM66	P	JAK/STAT pathway	TRIM66 promoted STAT2 and IL-2 and cell proliferation and migration in prostate cancer cells.
Miyajima [128]	Cancer Res, 2008	TRIM68	P	AR, TIP60, p300	TRIM68 regulates AR-mediated transcription. Knockdown of TRIM68 inhibited colony formation of LNCaP cells.
Li [129]	Epigenetics, 2012	TRIM68	P	miR29a and miR-1256	Isoflavone increased the levels of miR29a and miR-1256, which resulted in a decreased expression of TRIM68, thereby inhibiting cell growth and invasion.
Nie [130]	Eur J Med Res, 2023	TRIM68	P	YTHDF1	YTHDF1 promotes cancer progression by regulating TRIM68 in vitro and in vivo.

NA: data not available, TRIM: tripartite motif, EMT: epithelial–mesenchymal transition, IHC: immunohistochemistry.

## Data Availability

The data presented in this study are available in this article and Supplementary Materials.

## Supplementary Materials

The following supporting information can be downloaded at: https://www.mdpi.com/article/10.3390/cancers17142367/s1, Table S1: PRISMA 2020 Checklist; Table S2: Risk of bias assessment for NRCTs (ROBINS-I).
