# Supplementary material for: The Roles of Tripartite Motif Proteins in Urological Cancers: A Systematic Review
Source: Cancers (Basel). 2025 Jul 16;17(14):2367. doi: 10.3390/cancers17142367 (PMC12294046; doi:10.3390/cancers17142367)
Supplement: Supplementary file 1 [file cancers-17-02367-s001.zip › Table S2.pdf]

Table S2. Risk of bias assessment for NRCTs (ROBINS-I).

Study	Year	Confounding	Paticipants' selection	Classification of interventions	Deviations from intended intervention	Missing data	Measurement of outcomes	Selection of the reported result	Overall
Zheng	2022	High	High	Low	Low	High	High	High	High
Xiao	2018	High	High	Low	Low	High	High	High	High
Wei	2020	High	High	Low	Low	High	High	High	High
Shen	2022	High	High	Low	Low	High	High	High	High
Yuan	2022	High	High	Low	Low	High	High	High	High
Wu	2020	High	High	Low	Low	High	High	High	High
Caratozzolo	2014	High	High	Low	Low	High	High	High	High
Li	2020	High	High	Low	Low	High	High	High	High
Lin	2014	High	High	Low	Low	High	High	High	High
Simoni	2024	High	High	Low	Low	High	High	High	High
Chen	2021	High	High	Low	Low	High	High	High	High
Jiang	2020	High	High	Low	Low	High	High	High	High
Yu	2020	High	High	Low	Low	Moderate	Moderate	High	Moderate
Zheng	2024	High	High	Low	Low	High	High	High	High
Xiao	2021	High	High	Low	Low	High	High	High	High
Song	2023	High	High	Low	Low	High	High	High	High
Jingushi	2015	High	High	Low	Low	High	High	High	High
Xu	2020	High	High	Low	Low	High	High	High	High
Miao	2021	High	High	Low	Low	High	Moderate	High	Moderate
Yamada	2020	High	High	Low	Low	High	High	High	High
Ren	2021	High	High	Low	Low	High	High	High	High
Chen	2021	High	High	Low	Low	High	High	High	High
Gan	2021	High	High	Low	Low	High	High	High	High
Hu	2017	High	High	Low	Low	High	High	High	High

Wang	2021	High	High	Low	Low	High	High	High	High
Zhang	2024	High	High	Low	Low	High	High	High	High
Zhang	2023	High	High	Low	Low	High	High	High	High
He	2003	High	High	Low	Low	High	High	High	High
Xue	2010	High	High	Low	Low	High	High	High	High
Li	2006	High	High	Low	Low	High	High	High	High
Tsai	2019	High	High	Low	Low	High	High	High	High
Deng	2021	High	High	Low	Low	High	High	High	High
Xiao	2023	High	High	Low	Low	High	High	High	High
Xue	2015	High	High	Low	Low	High	High	High	High
Agarwal	2021	High	Moderate	Low	Low	High	High	Moderate	Moderate
Tang	2022	High	High	Low	Low	High	High	High	High
Xie	2021	High	High	Low	Low	High	High	High	High
Palmbos	2023	High	High	Low	Low	High	High	High	High
Zhang	2017	High	High	Low	Low	High	High	High	High
Wang	2021	High	High	Low	Low	High	High	High	High
Chen	2017	High	High	Low	Low	High	High	High	High
Qiu	2024	High	High	Low	Low	High	High	High	High
Wei	2018	High	High	Low	Low	High	High	High	High
Xiao	2023	High	High	Low	Low	High	High	High	High
Chen	2021	High	High	High	High	High	High	High	High
Offermann	2021	High	High	Low	Low	High	High	High	High
Pan	2019	High	High	Low	Low	High	High	High	High
Pan	2023	High	High	Low	Low	High	High	High	High
Guo	2022	High	High	Low	Low	High	High	High	High
Spirina	2020	High	High	Low	Low	High	High	High	High
Buczek	2016	High	High	Low	Low	High	High	High	High
Birch	2014	High	Moderate	Low	Low	High	High	High	High
Chatterjee	2013	High	High	Low	Low	High	High	High	High

Zhang	2003	High	High	Low	Low	High	High	High	High
He	1997	High	High	Low	Low	High	High	High	High
Yang	2004	High	High	Low	Low	High	High	High	High
Höflmayer	2021	High	High	Low	Low	High	High	High	High
Bai	2021	High	High	Low	Low	High	High	High	High
Guan	2019	High	High	Low	Low	High	High	High	High
Wang	2016	High	High	Low	Low	High	High	High	High
Takayama	2018	High	High	Low	Low	High	High	High	High
Li	2022	High	High	Low	Low	High	High	High	High
Yu	2022	High	High	Low	Low	High	High	High	High
Fong	2018	High	Moderate	Low	Low	High	Moderate	High	Moderate
Xue	2024	High	High	Low	Low	High	High	High	High
Kanno	2014	High	High	Low	Low	High	High	High	High
Zhou	2023	High	High	Low	Low	High	High	High	High
Chen	2022	High	High	Low	Low	High	High	High	High
Fujimura	2014	High	High	Low	Low	High	High	High	High
Kimura	2018	High	High	Low	Low	High	High	High	High
Zhao	2023	High	High	Low	Low	High	High	High	High
Tan	2017	High	High	Low	Low	High	High	High	High
Li	2021	High	High	Low	Low	High	High	High	High
Zhou	2023	High	High	Low	Low	High	High	High	High
Fujimura	2016	High	High	Low	Low	High	High	High	High
Lin	2016	High	High	Low	Low	High	High	High	High
Fan	2023	High	High	Low	Low	High	High	High	High
Ma	2022	High	High	Low	Low	High	High	High	High
Cao	2020	High	High	Low	Low	High	High	High	High
Miyajima	2008	High	High	Low	Low	High	High	High	High
Li	2012	High	High	Low	Low	High	High	High	High
Nie	2023	High	High	Low	Low	High	Moderate	High	High

NRCTs: non-randomized comparative studies, ROBINS-I: Risk Of Bias In Non-Randomized Studies -of Interventions.
